# Generative artificial intelligence (GenAI) use and dependence: an approach from behavioral economics

**DOI:** 10.3389/fpubh.2025.1634121

**Published:** 2025-08-06

**Authors:** Oscar Robayo-Pinzon, Sandra Rojas-Berrio, Jorge E. Camargo, Gordon R. Foxall

**Affiliations:** ^1^School of Management and Business, Universidad del Rosario, Bogotá, Colombia; ^2^Faculty of Economics, Universidad Nacional de Colombia, Bogotá, Colombia; ^3^Faculty of Engineering, Universidad Nacional de Colombia, Bogotá, Colombia; ^4^Cardiff Business School, Cardiff University, Cardiff, United Kingdom; ^5^School of Management, Reykjavik University, Reykjavik, Iceland

**Keywords:** GenAI, dependence, temporal discounting, public mental health, economy of attention, digital well-being, behavioral economics

## Abstract

**Objective:**

This study aims to explore the perceived dependence on Generative Artificial Intelligence (GenAI) tools among young adults and examine the relative reinforcing value of AI chatbots use compared to monetary rewards, applying a behavioral economics approach.

**Participants/methods:**

A total of 420 university students from Bogotá, Colombia, participated in an online survey. The study employed a Multiple Choice Procedure (MCP) to assess the relative reinforcement between different durations of GenAI use (1, 2, and 4 weeks) and monetary rewards, which varied in amount and delay. Additionally, an adapted AI Dependence Scale evaluated levels of dependence on AI tools. Data analysis included repeated measures ANOVA to examine the effects of reward magnitude and delay on choices, and correlations to assess the relationship between perceived dependence and reinforcement values.

**Results:**

Participants reported low average dependence on AI tools (mean AI Dependence Scale score = 65.6), with no significant gender differences. MCP findings indicated significant differences in crossover points across varying durations or delays for AI chatbots use, suggesting a higher relative value of use for the option to use AI chatbots immediately. The average reinforcement value for AI use versus monetary rewards did significantly vary with reward magnitude. On the other hand, significant differences were found in the levels of perceived dependence on AI, according to the average daily time of AI tool use.

**Conclusion:**

The results suggest that young adults exhibit low perceived dependence on GenAI tools but show differential reinforcement values based on usage duration or delay conditions. This behavioral economics approach provides novel insights into decision-making patterns related to AI chatbots use, emphasizing the need for further research to understand the psychological and social factors influencing dependence on AI technologies.

## Introduction

Generative artificial intelligence (GenAI) refers to AI systems capable of producing novel content and solutions, including images, dialogs, videos, narratives, and music. GenAI’s adaptability enables it to learn both human and programming languages, as well as advanced concepts in fields such as art, chemistry, and biology, thereby allowing it to recombine information to address new challenges (1). Projections indicate that GenAI will experience an annual growth rate of 36% through 2030, accounting for 55% of the AI software market. By 2030, investments in specialized GenAI applications are expected to reach $79 billion per year in sectors like healthcare, security, and content marketing (2). In terms of user base, a 131% global increase is anticipated between 2024 and 2030, reaching 729 million active users and a market value of $365 billion (3). However, the pace of GenAI adoption varies by region, with a recent study reporting usage rates of 73% in India, 45% in the United States, and 29% in the United Kingdom (4).

Academic literature has focused mainly on GenAI adoption in higher education, recognizing its dual role as a tool for students and teachers and as a transformative force within educational environments (5). Research highlights both the potential benefits and risks associated with GenAI. Advantages include the provision of personalized online resources—such as books, journals, and videos—as well as the use of virtual reality applications and simulators across various disciplines (6). Nonetheless, as GenAI adoption accelerates, concerns are emerging regarding its negative impacts, particularly among younger users. One key issue is the potential for technological dependence, defined as a high level of user reliance on a technological service to the extent that it becomes indispensable for achieving objectives (7). While initial benefits may include increased efficiency in routine tasks, long-term consequences could involve diminished autonomous learning and reduced psychological resilience (8).

The user experience of GenAI tools, especially chatbots like ChatGPT, is central to understanding these concerns. Chatbots simulate fluid conversations, delivering responses in text, voice, images, or videos across diverse knowledge domains (9). The highly interactive and personalized nature of chatbots—characterized by human-like conversation, assertive language, and the expression of emotions and opinions—has raised concerns about their potential to foster technological dependence. Unlike social media networks, which have been previously linked to digital dependence, chatbots offer a more intimate and personalized user experience, lacking the broader social interaction typical of social media. Therefore, it is necessary to adapt and validate models originally developed to study social media dependence to the context of GenAI-based chatbot ecosystem (10).

Theoretical research on the use of digital platforms has been extensive, leading to a gray area regarding the conceptual definitions of their potential negative impacts. This ambiguity is evident in the use of terms such as dependence and addiction, for which clear distinctions have yet to be established. In this regard, some scholars classify technological addiction as a form of non-substance or behavioral addiction (11). This category encompasses disorders related to online gambling, shopping, and, more recently, social media network (SMN) addiction (12). SMN addiction has been linked to adverse outcomes such as depressive symptoms (13, 14), sleep disturbances (15), and negative impacts on self-image and self-esteem (16), with adolescents and young adults being particularly susceptible (17).

On the other hand, recent research has begun to document evidence of dependence on AI-based technologies among young adults. For example, a study of Chinese university students found that increased frequency of GenAI use correlates with greater confidence and perceived learning efficiency, but also with higher dependence levels (7). Another study identified a significant intention among university students to adopt technologies such as teacher bots (T-bots), with interactivity, perceived intelligence, and anthropomorphism cited as facilitating factors (18). Further findings suggest that continued GenAI use may influence emotional well-being: emotional attachment, rather than functional attachment, positively predicts technological addiction to GenAI, with perceived empathy and anthropomorphism being key contributors to emotional attachment (10).

Despite these insights, most studies on GenAI dependence and potential technological addiction among young users have employed cognitive and perceptual approaches, typically using adapted scales from research on SMN use. This highlights the need for alternative theoretical perspectives to deepen understanding of this phenomenon (7, 19). Behavioral economics—a field integrating microeconomics and operant psychology—has recently been applied to the study of dependence on digital technologies such as SMN (20). In particular, the concept of temporal discounting has been employed to examine correlations between impulsivity (as measured by scales or intertemporal choice tasks) and technology dependence or usage duration (21, 22).

Behavioral economics provides a theoretical framework for understanding choice behavior and decision-making by examining how individuals allocate activities among concurrently available reinforcers (23). Within this framework, the Multiple-Choice Procedure (MCP) is a method designed to assess the interaction between drug use and substitute reinforcers, thereby measuring the relative value of a substance or behavior in terms of reinforcement (24). The MCP is grounded in the idea that the relative reinforcing value of a substance or behavior is influenced by both intrinsic qualities and environmental factors, including the availability of the substance and the presence of alternative reinforcers (25).

In practice, the MCP presents participants with structured choices between a target behavior (e.g., psychoactive substance use or GenAI tool utilization) and an alternative reinforcer (such as monetary compensation) that increases progressively in magnitude. The aim is to identify the crossover point—the condition at which the participant shifts preference from the target behavior to the alternative reinforcer (26). The crossover point thus reflects the relative reinforcement value of the target behavior: a higher crossover point indicates a greater reinforcement value, as the participant requires a larger monetary incentive to forgo the target activity (27, 28). This approach is underpinned by the concept of delay discounting, which refers to the devaluation of future outcomes in decision-making processes (29). As the delay to a consequence increases, its influence on current decisions diminishes, forcing individuals to choose between an immediate reward and a discounted delayed reward (30).

These intertemporal choices, shaped by the magnitude of available incentives, are central to decision-making involving outcomes at different points in time. The magnitude effect predicts that individuals are more likely to wait for larger rewards than for smaller ones (31–33). This dynamic compels individuals to choose between a larger, later reward (LL) and a smaller, sooner reward (SS) (34). For example, a person might decide between investing money for future gain (LL) or spending it immediately on a new technology (SS), or between saving for a desired product (LL) and making an impulsive purchase (SS). Empirical evidence suggests that most people tend to prefer SS over LL (35).

Applying a behavioral economics perspective to the study of GenAI use can enhance understanding of usage patterns and perceived dependence among young adults, complementing existing cognitive-focused research. Recent studies have primarily addressed dependence on smartphones (36, 37) and SMNs (38–41). However, no prior research has used behavioral economics or the MCP to determine the relative reinforcement values of monetary incentives versus GenAI use over varying time periods. Considering these gaps, the present study addresses the following research questions: (RQ1) What is the relative reinforcing value of monetary incentives compared to varying durations of GenAI use? (RQ2) What is the relationship between the relative use value of GenAI and perceived dependence on GenAI?

## Materials and methods

### Participants

This research had participants from the main universities in Bogotá (Colombia), and gift cards were offered as an incentive for answering the online questionnaire. The final sample comprised 420 participants: 39.1% identify as male (mean age = 23.6 years, SD = 6.2, range = 16–67); 58.0% as female (mean age = 22.6 years, SD = 5.8, range = 17–57); 1.4% as non-binary gender; and 1.4% preferred not to say. Of the participants, 87.6% had a bachelor’s degree or were enrolled in an undergraduate program. The socioeconomic distribution included 24.69% of households from the middle socioeconomic stratum, 27.9% from the lower-middle stratum, 24.3% from the lower stratum, 14.5% from the upper-middle stratum, 2.9% from the upper stratum, and 5.5% from the lowest stratum. Table 1 presents the basic measures of the age of the participants. Data collection was conducted through multiple waves of online survey distribution via institutional email, following approval of the instrument by the Ethics Committee of Universidad del Rosario. Eligibility criteria required participants to be frequent users of artificial intelligence tools with prior experience; individuals with no or only occasional use of such technologies were excluded. This recruitment strategy ensured the relevance and robustness of the responses in relation to the study’s objectives.

**Table 1:** Measures for age of participants.

Sex	M	S	Min	Max
Male	23.6	6.23	16	67
Female	22.6	5.80	17	57

### Instruments

#### Multiple choice procedure

The MCP consists of a series of situations in which participants must choose in a hypothetical context between certain amounts of AI application usage time (ChatGPT, Gemini, Perplexity AI, Bing Chat, Claude 3.) and different monetary rewards in Colombian pesos (COP) (26, 42, 43). Each situation poses the following scenario before the questions with the two discrete choice options appear: “*Next, you will have to answer several questions in which you will choose between being able to use Artificial Intelligence (AI) applications (*e.g.*, ChatGPT, Gemini, Perplexity AI, Bing Chat, Claude, etc.) for a week OR immediately receive a certain amount of money (in Colombian pesos) in exchange for not being able to use these applications for a week*.” Below is an example of one of the five discrete choice questions that made up each situation.

*Which alternative do you prefer?*

- *To be able to use AI applications for a week.*
- *Receive $10,000 immediately in exchange for NOT using AI apps for a week.*

Participants completed situations 1 to 6 of the MCP, which included three levels of AI application usage time (1, 2 and 4 weeks) and two options for the time to receive the monetary reward (immediately or with a 3-month delay). The durations of AI application usage were selected from a pilot test conducted by the researchers with a sample of 20 participants. Cognitive validation identified the different categories of duration based on responses to open-ended questions that probed the frequency of use of AI tools. Each amount of reinforcement was presented sequentially, with pairings of 1, 2 and 4 weeks versus immediate money and 1, 2 and 4 weeks versus receiving money after 3 months. The monetary reward started at 10,000 COP (approximately US$2.41) and increased to 25,000 COP; 50,000 COP; 75,000 COP; and finally, 100,000 COP (approx. US$24.12). These amounts were also obtained from the pilot test mentioned above. Each situation generated a unique crossover point to reflect the comparative reinforcement value of the AI application usage versus the reinforcement value of the respective monetary reward. Every participant answered 30 discrete-choice questions, which corresponded to five options for each of the six MCP versions. Each version produced a singular crossover point, representing the primary data, outlined as the relative reinforcement value of utilizing AI applications vs. an alternative monetary reinforcement. After that, situation 7 involved hypothetical scenarios between the participant’s choice of whether to give up his or her personal information in exchange for using the premium version of the preferred AI application for a period of 1 year.

#### AI dependence scale

Social Media Addiction Scale (SMAS) was developed by (44), from a sample of 775 college students with social media applications (Facebook, Twitter and/or Instagram). The study sought to develop a reliable and valid measurement tool to measure social media addiction. In this research, the SMAS was adapted for the context of AI application usage. To carry out the adaptation, the scale was first back-translated and the wording of the items was adjusted within the context of the use of AI tools. Then, a cognitive validation pilot test was developed with a sample of 20 university students. In the process, the original number of scale items (32 items) was maintained. Total AI Dependence Scale scores, which resulted from the sum of the scores for each item, were used in the analyses.

### Procedure and data analysis

Participants completed the online questionnaire using the Google Forms tool. The questionnaire was structured as follows: (I) Demographic questions, (II) Use of AI apps, (III) Discrete choice questions (Situations 1 to 7), and (IV) Scale of attitudes toward the use of AI chatbot applications. The statistical analyses were conducted after verifying the relevant assumptions for each test performed. For parametric analyses, assumptions of normality and homoscedasticity were assessed using the Shapiro–Wilk test and Levene’s test, respectively. When these assumptions were not met, non-parametric alternatives were used. Effect sizes were calculated and reported alongside *p*-values to enhance the interpretability of the results. Importantly, the online questionnaire was configured to require complete responses, which prevented the submission of incomplete surveys. As a result, there was no missing data in the final dataset, and all cases included in the analysis were complete.

## Results

32.61% of the participants reported that they use AI tools between 1 and 2 h a day; 15.95% between 2 and 3 h; 10.23% for 4 h or more; and the remaining 41.19% use them for 1 h or less per day. Most of the participants reported that the AI tools they use the most is ChatGPT (74.7%), followed by Copilot (8.1%) and Gemini (7.4%). Table 2 presents the percentages of use of the AI tools in relation to usage time.

**Table 2:** AI tools usage time.

1 h or less	4 h or more	Between 1 and 2 h	Between 2 and 3 h
0.4119048	0.1023810	0.3261905	0.1595238

Table 3 shows the results obtained from three questions related to the intention to share progressively more sensitive personal information in exchange for a one-year subscription to the professional version of their favorite AI chatbot (e.g., ChatGPT). First, it is observed that a very significant percentage of participants (65.7%) would be willing to share their email address, age, gender, and cell phone number in exchange for this benefit, followed by a considerable 17.1% who would share their digital footprint (the IP address of their devices, information about the network they connect to, the websites they visit, and the apps they use). Finally, 12.1% would be willing to share their biometric information (iris scan and facial recognition scan) in exchange for this one-year subscription.

**Table 3:** Disposition to share personal data, digital footprint, and biometric data to gain access to the AI tool premium version for a year.

Type of data	Yes	No	Total
Personal Data	65.7%	34.3%	100%
Digital Footprint	17.1%	82.9%	100%
Biometric Data	12.1%	87.9%	100%

**Figure 1:**
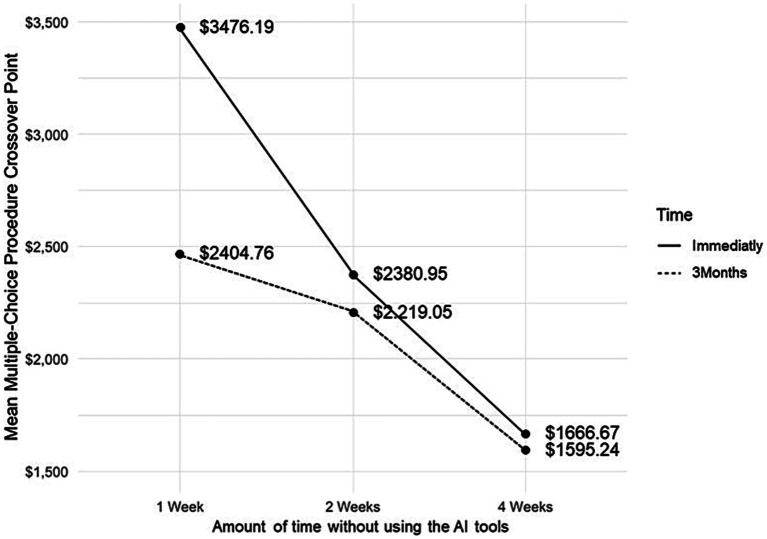
Mean multiple choice procedure crossover points of each of the six versions.

The results in the AI Dependence Scale indicate that on average the participants have a low level of dependence on the use of AI tools (M = 64.34, SD = 17.31). The average level obtained is considered to be low as the maximum score a participant can obtain is 160 (32 items x 5, assuming a coding of 5 for the answer option ‘always’). Furthermore, as can be seen in Table 4, there is no significant differences in the crossover points of the six versions of the MCP, nor is there a significant difference in the AI Dependence Scale according to the six versions of the MCP. To determine whether the MCP was affected by the size of the incentive and/or delay while assessing hypothetical decisions between using AI tools and an alternate financial reward, a 2 × 3 repeated measures factorial ANOVA (Delay [immediately or with a 3-month delay] Magnitude [1, 2, 4 weeks to use AI tools]) was conducted, using MCP crossover points as the dependent variable. The separate models for men and women yielded non-significant results. The models separated by AI tools yielded non-significant results. Table 5 displays only the model for the entire sample. Table 6 and Figure 1 show the average crossover point values for the six MCP versions.

**Table 4:** Bivariate correlations.

Variable	MCP1	MCP2	MCP3	MCP4	MCP5	MCP6	MCP_PROM	AI dependence scale
MCP1	1.00	0.69	0.52	0.64	0.59	0.47	0.79	−0.10
MCP2	0.69	1.00	0.76	0.65	0.72	0.64	0.88	−0.07
MCP3	0.52	0.76	1.00	0.65	0.69	0.77	0.85	−0.06
MCP4	0.64	0.65	0.65	1.00	0.80	0.67	0.87	−0.01
MCP5	0.59	0.72	0.69	0.80	1.00	0.78	0.89	−0.01
MCP6	0.47	0.64	0.77	0.67	0.78	1.00	0.83	−0.01
MCP_PROM	0.79	0.88	0.85	0.87	0.89	0.83	1.00	−0.05
AI dependence scale	−0.10	−0.07	−0.06	−0.01	−0.01	−0.01	−0.05	1.00

**Table 5:** Factorial analysis of variance for repeated measures.

Effect	DFn	DFd	F	*p*	*p* < 0.05	Ges
1. Magnitude	2.00	822.00	39.82	0.00	*	0.04
2. Time	1.00	411.00	18.17	0.00	*	0.00
3. Magnitude: Time	2.00	822.00	28.68	0.00	*	0.03

*Statistically significant *p*-value.

**Table 6:** Means and standard deviations for the main study variables.

Variable	Full sample (*N* = 420)		Males (*n* = 168)		Females (*n* = 252)		*Tval*
	*M*	*SD*	*M*	*SD*	*M*	*SD*	
MCP1	3476.19	4767.82	2882.35	4542.79	3880.00	4882.72	0.03
MCP2	2380.95	4264.26	1941.18	3966.88	2680.00	4438.06	0.08
MCP3	1666.67	3731.22	1470.59	3552.11	1800.00	3849.58	0.37
MCP4	2404.76	4278.82	1941.18	3966.88	2720.00	4458.83	0.07
MCP5	2119.05	4091.45	1823.53	3872.76	2320.00	4229.56	0.21
MCP6	1595.24	3666.01	1470.59	3552.11	1680.00	3746.16	0.56
Total	64.34	17.31	63.75	17.04	64.74	17.51	0.56

**Figure 2:**
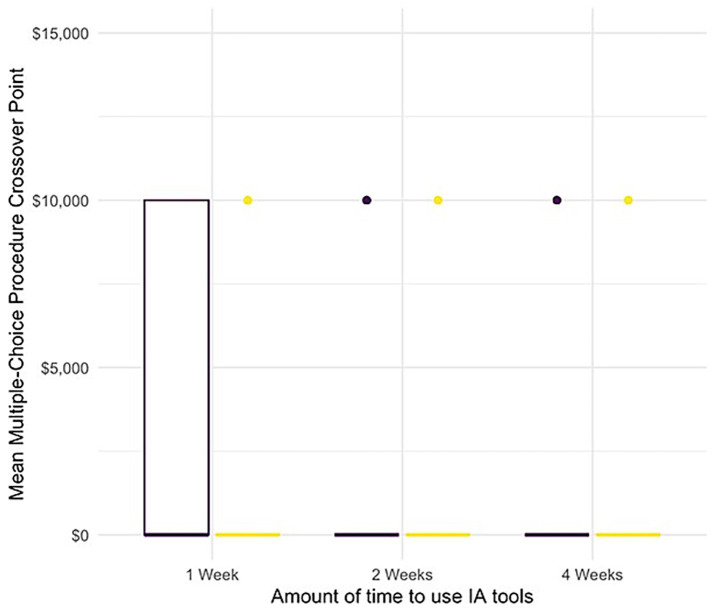
Differences in the average crossover points according to the magnitude of the reinforcer.

The results of the factorial ANOVA (see Table 5) indicate that the amount of the monetary reward (*F*_(2, 822)_ = 39.82, *p* < 0.05), the delay of the alternative reinforcer (*F*_(1, 411)_ = 18.17, *p* < 0.05), and the interaction between these two factors (*F*_(2, 822)_ = 28.68, *p* < 0.05), all have statistically significant effects. Figure 2 illustrates these results.

Table 4 indicates that there is no statistically significant relationship between the mean crossover point of the MCP and the total AI Dependence Scale scores. On the other hand, the analysis of variance (ANOVA) shows that the participants’ usage of each period has a statistically significant impact (F_(2, 822)_ = 28.68, *p* < 0.05).

Finally, based on ANOVA and Bonferroni tests, significant differences were observed in the average scores of AI dependence across groups defined by the average daily AI tool usage time. Specifically, participants who reported using AI tools for between 2 and 3 h daily had significantly higher average scores than those using them for between 1 and 2 h and those using them for less than 1 h. Additionally, participants reporting usage of 4 h or more did not have significant differences in average scores compared to those with usage times between 1 and 2 h or less than 1 h (See Table 7).

**Table 7:** ANOVA between AI Dependence scale scores and time of daily use of AI tools.

Df	Sum Sq	Mean Sq	*F* value	Pr (>F)
3	3380.80	1126.93	3.838	0.009*
416	122143.18	293.61		

*Statistically significant *p*-value.

## Discussion

This study aimed to determine the relative usage value of GenAI tools among young adult users and to explore the relationship between this usage value and perceived dependence on this emerging digital technology. By applying behavioral economics as an alternative theoretical framework to the predominantly cognitive approaches in existing research, this work contributes to a broader understanding of dependence on new digital technologies—a topic of increasing relevance for public mental health and digital well-being management.

The findings indicate that participants generally spend less time per day using AI tools compared to the time reported for social media networks (SMNs) in previous studies (38). Specifically, 41.19% of participants reported using AI tools for 1 hour or less per day, whereas 40% reported using SMNs for 1 to 2 h daily, with 38% using them for 2 to 3 h and 16% for 4 h or more. In contrast, only 15.95% of participants used AI tools for 2 to 3 h, and 10.23% for more than 4 h per day. This suggests a different time allocation pattern between the two technologies, likely reflecting the more functional, work- or academically-oriented use of GenAI, as opposed to the entertainment-driven use of SMNs. Additionally, GenAI chatbots like ChatGPT offer highly interactive features, distinguishing them from current SMNs. These differences warrant further investigation in future studies.

With respect to the results obtained from applying the MCP to the context of AI tool usage, significant differences were observed for the magnitude of the hypothetical monetary incentive, the delay period, and the interaction between these two factors. These findings are generally consistent with previous literature employing the MCP as a research methodology in non-substance addiction contexts, such as video games, gambling, and, more recently, social media networks (SMNs) (38, 42, 43, 45). When interpreting these results, it is important to note that, unlike prior studies on behavioral addictions—where the imposed waiting periods for abstaining from the behavior typically ranged from a few minutes to a few hours—in this study, the waiting periods for refraining from AI tool use ranged from 1 to 4 weeks.

This qualitative distinction between types of digital behaviors, particularly when comparing AI tool use to social media networks (SMNs), is reflected in the observed trends. Previous studies have shown that as participants are required to wait longer before engaging in a target behavior—such as using SMNs—they expect higher amounts of hypothetical monetary compensation. Moreover, if the monetary reward is delayed, the required amounts at the crossover point also increase. In contrast, for AI tool use, the magnitude of monetary incentives decreases as the waiting period without access to these tools extends from 1 to 4 weeks. This decrease occurs for both immediate and three-month delayed delivery of the monetary reward. The statistically significant results from the ANOVA test further confirm this dual effect.

One possible explanation for this pattern in choice behavior across the six MCP scenarios may relate to the specific benefits provided by AI chatbots. As noted by (7), increased use of these tools enhances perceived confidence and efficiency, but also heightens dependency. Consequently, their value may be primarily associated with immediate utility, prompting participants to demand higher compensation for abstaining from use over a short-term period, such as 1 week. As the waiting period increases to 2 or 4 weeks, the monetary value participants require decreases for both reward delivery conditions, though it remains higher for immediate delivery.

This finding may indicate a qualitative difference between SMNs—which are used frequently throughout daily life—and AI tools, which have not yet achieved the same level of omnipresence in users’ daily routines. As a result, participants likely perceive longer future periods without AI chatbot use as less significant than immediate restrictions. This aspect warrants further investigation. In relation to research question 1, the study confirms the relative reinforcing value of AI technologies and their sensitivity to both the magnitude of monetary incentives and the timing of reward delivery, as well as their interaction (see Table 5). These results are consistent with prior research (42).

In addressing the second research question, the analyses reveal a significant association between scores on the AI Dependence Scale and the categories of AI tools used daily (see Table 7), consistent with previous research (43, 46). Notably, individuals who reported using GenAI tools for 2 to 3 h per day had substantially higher dependence scores than those using them for 1 to 2 h or less. Unexpectedly, those who reported using AI tools for 4 h or more did not significantly differ in dependence scores from those with lower usage times. This finding suggests that very frequent users may have normalized their interaction with AI chatbots, perceiving them less as a problematic behavior and more as integral companions or assistants in their daily routines. This phenomenon merits further investigation to understand the nuances of user perception and technological integration.

From a public mental health perspective, although the present study found generally low levels of perceived dependence on AI chatbots, existing literature indicates that factors such as emotional attachment could contribute to increased dependence over time (10). The distinctive features of AI chatbots—including their capacity to understand users and deliver natural, human-like responses—may foster a sense of intimacy and closeness, potentially elevating emotional reliance. In light of interventions for other forms of behavioral addictions (36, 47), it is recommended that technology companies developing AI-based tools implement responsible use features, such as daily usage reports and alerts for excessive use. Furthermore, these platforms should offer AI literacy modules to inform users about the underlying mechanisms and potential risks of these technologies. Such measures would support informed and healthy engagement with AI tools, contributing to the promotion of digital well-being and responsible technology use.

This article presents an initial exploration of the MCP in relation to AI tool usage along with empirical evidence supporting the applicability of behavioral economics to the digital consumption patterns of young users (48). These findings facilitate the development of potential therapeutic and societal treatment approaches in response to this escalating consumption phenomena.

### Limitations and future research

Some limitations must be considered. First, there was no use of placebo control settings. Second, the results should be cautiously extrapolated because the sample consisted of college students. Because AI tools usage varies between regions, future research should consider a more varied sample and, ideally, compare populations from different territories (4). Furthermore, future research should explore replicating this study within a longitudinal intervention in a natural environment, thereby enhancing ecological validity to complement existing experimental studies. By broadening the context to natural environments, rather than laboratory or experimental settings, The MCP can be established as an alternative methodology grounded in behavioral economics that aids in addressing dependence behaviors on these AI-based interactive technologies.

Future research should explore the differences in usage motives and user-perceived benefits between different digital technologies present in users’ daily lives, particularly between SMNs and AI chatbots. In the near future researchers should also start considering AI agents or assistants as a new form of this technology that, possessing even stronger attributes of anthropomorphisation, empathy and emotion expression, could reinforce the emerging patterns of dependence on these technologies. As the use of chatbots, or even more sophisticated AI agents, come to reach a greater share of daily digital consumption time, researchers may find intertemporal choice patterns closer to those reported for SMN’s, as the digital platforms most used daily by young adults today. These possibilities could be the subject of study in the short and medium term. Finally, studies are needed to empirically validate interventions based on behavioral economics principles aimed at increasing levels of AI literacy, detecting patterns of use that may be problematic, as well as considering emotional factors in the evolution of users’ dependence on these digital tools.

## Data Availability

The raw data supporting the conclusions of this article will be made available by the authors, without undue reservation.
